# Ovarian thecoma presenting with acute ovarian torsion in pregnancy; report of a rare case

**DOI:** 10.1002/ccr3.5986

**Published:** 2022-06-26

**Authors:** Asieh Maleki, Maedeh Khosravi, Azaadeh Masrouri

**Affiliations:** ^1^ Obstetrics and Gynecology, Omolbanin Hospital Mashhad University of Medical Sciences Mashhad Iran; ^2^ Women's Reproductive Health Research Center Mashhad University of Medical Sciences Mashhad Iran

**Keywords:** ovarian tumor, pregnancy, thecoma

## Abstract

Thecoma is a commonly benign ovarian tumor of the group “Sex cord‐stromal neoplasms.” This group represents <5 percent of ovarian tumors. Thecoma is extremely rare in pregnancy. Here, we describe a 17‐week pregnant woman presenting with acute ovarian pedicle torsion as a result of an ovarian thecoma.

## Graphical abstract

Thecoma is a type of sex cord‐stromal neoplasm, which is an infrequent ovarian tumor and extremely rare in pregnancy. It is important to consider such neoplasms in pregnant women suspected of adnexal mass, as the probability of ovarian tumor rupture or pedicle torsion increases during the course of pregnancy.

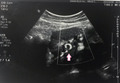

## 1 INTRODUCTION

Sex cord‐stromal neoplasms are consisted of <5 percent of ovarian tumors, and thecoma‐fibroma groups are an uncommon subdivision of these neoplasms. They are often benign tumors that arise from perifollicular stromal cells and mostly presented in postmenopausal women.^1^ It is exceedingly rare for thecoma to present in pregnancy and may be difficult to diagnose early, as the tumor growth and the physiologic increase in the uterus size during pregnancy are not simply differentiated. As the pregnancy advances and the uterus enlarges, the probability of ovarian tumor rupture or pedicle torsion increases. Prognosis is influenced by the gestational age and the size and histological features of the tumor.^2^ Here, we report a 17‐week pregnant woman presenting with acute abdominal pain as a result of an ovarian thecoma causing pedicle torsion.

## 2 CASE PRESENTATION

A 35‐year‐old gravida 3 para 0 (abortion 2) woman presented to the department of obstetrics emergencies with chief complaint of abdominal pain. The pain was predominantly in the hypogastric and right lower quadrant regions, had initiated a week earlier, and exacerbated since the night before. The patient expressed the presence of nausea and occasional vomiting since the beginning of pregnancy. She had no specific past medical history other than the two abortions (both at 6‐week gestational age, during the last year) and a surgical correction of ocular strabismus at the age of 22. She had regular menstruation of 28‐day cycles.

**FIGURE 1:**
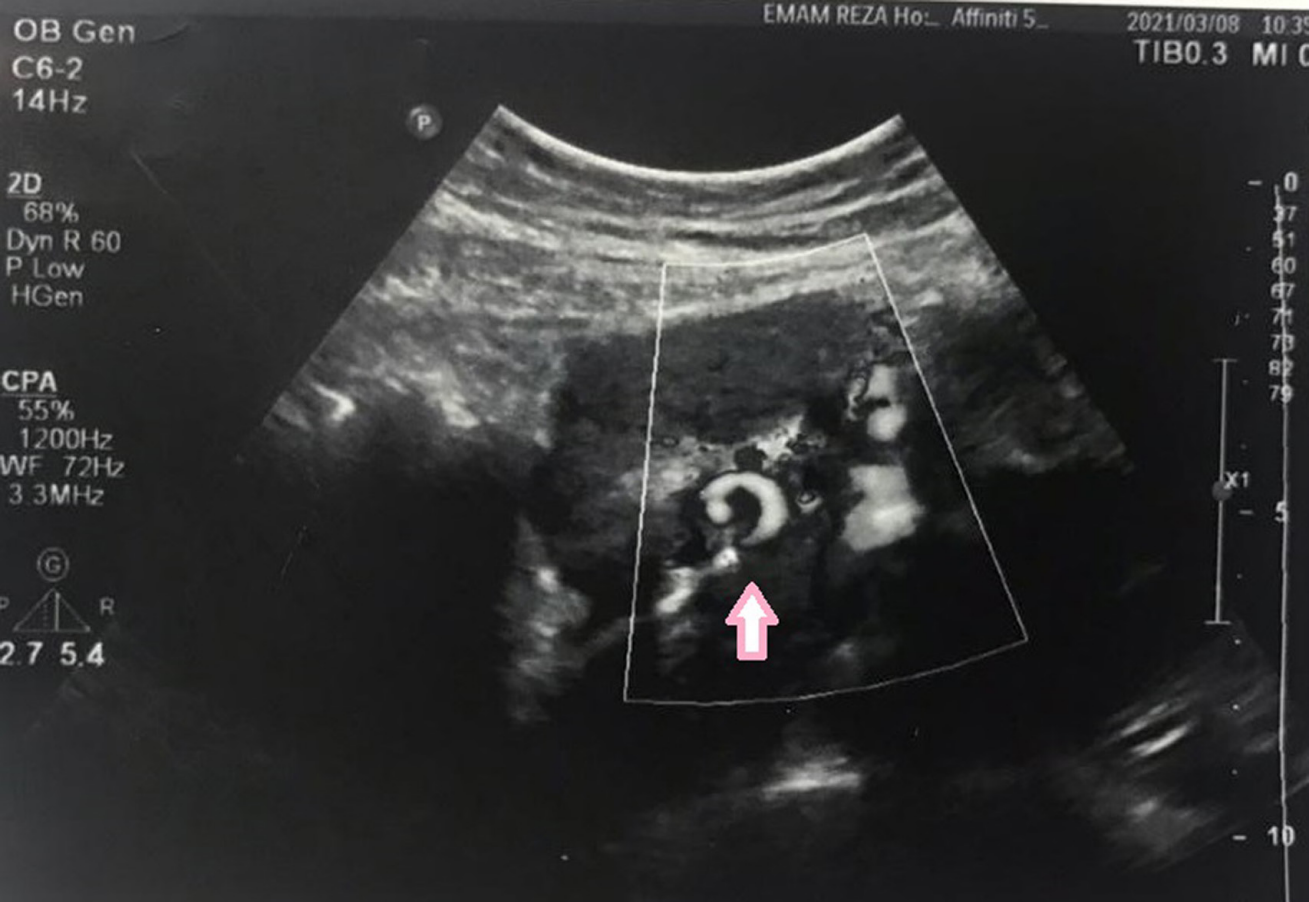
Whirlpool sign (pink arrow) which presented as twisted circular vessels on Doppler

On physical examination, the patient was relatively ill but not toxic, vital signs were stable, and abdominal examination revealed hypogastric tenderness. The patient was admitted and monitored in the emergency ward, and color Doppler ultrasound was requested with the initial diagnosis of ovarian torsion. Ultrasound study reported a normal 20 × 36 mm left ovary, and a 34 × 87 millimeter right adnexal mass, with whirlpool sign on the infero‐medial region (Figure 1). Due to the fact that the right ovary was not seen, the diagnosis of ovarian torsion was raised in the first place. Some fluid was also seen in the posterior cul‐de‐sac. The results of laboratory tests were as follows: complete blood test: WBC: 15.1, Hb: 9.1, Hct: 28.5, platelets: 245; BUN: 22, serum creatinine: 0.6; CRP (C‐reactive protein): 5.5 mg/L; and normal serum electrolytes. Tumor markers including CEA, CA125, and Alfa‐fetoprotein were all in normal range.

**FIGURE 2:**
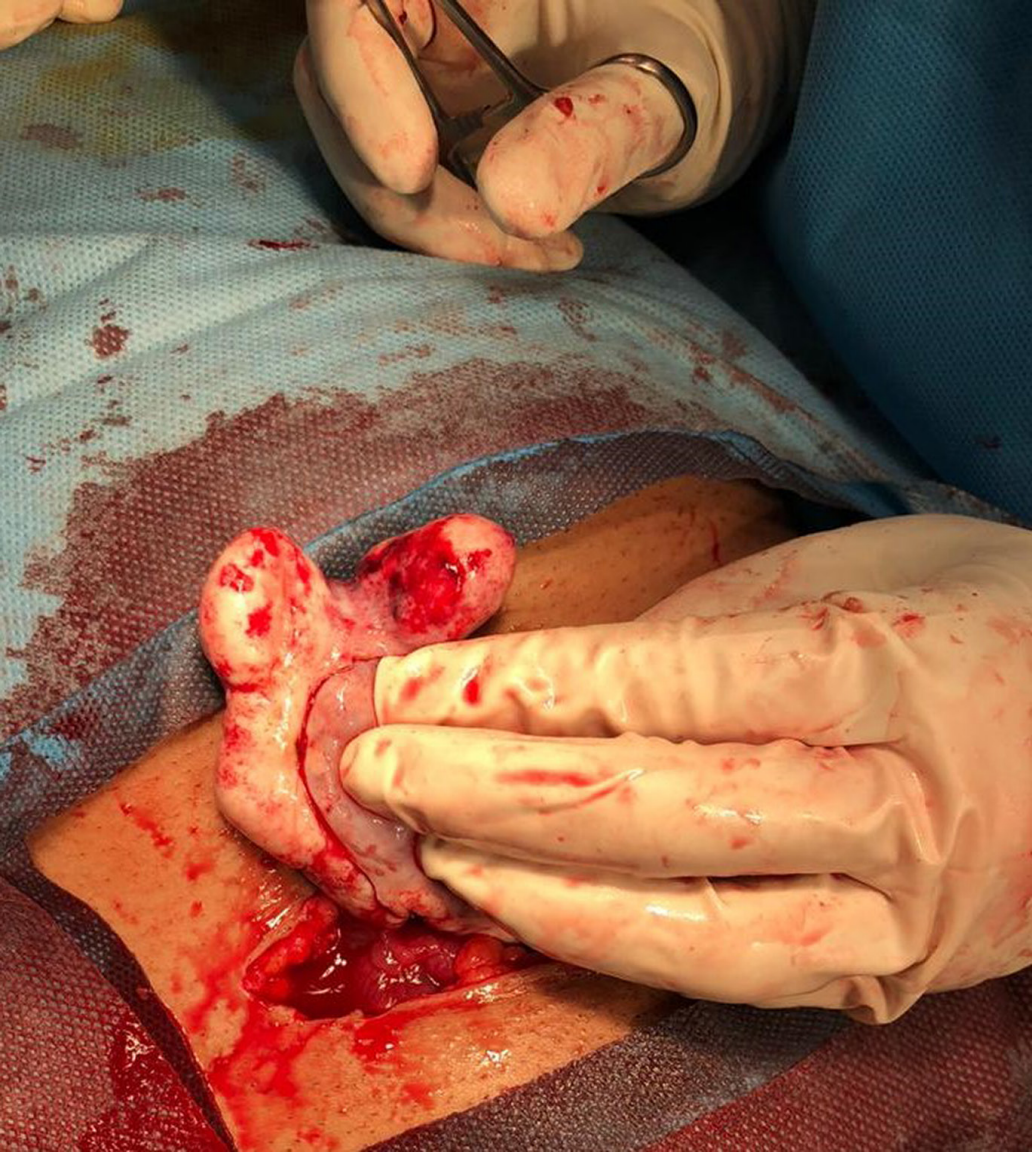
Intraoperative view of the ovarian mass causing pedicle torsion

The patient was transferred to the operating room, and a mini‐laparotomy incision was performed. A cyst with a white appearance and firm consistency was seen in the right ovary with three rotations. Tumor detorsion was first performed, and then the tumoral mass was resected and sent for pathologic examination (mass resection surgery; Figure 2). It is recommended for all women with thecoma to undergo pre or intraoperative endometrial sampling in order to rule out endometrial hyperplasia or carcinoma, but since our patient was pregnant, the “hands off the uterus” rule was followed.^3^, ^4^

**FIGURE 3:**
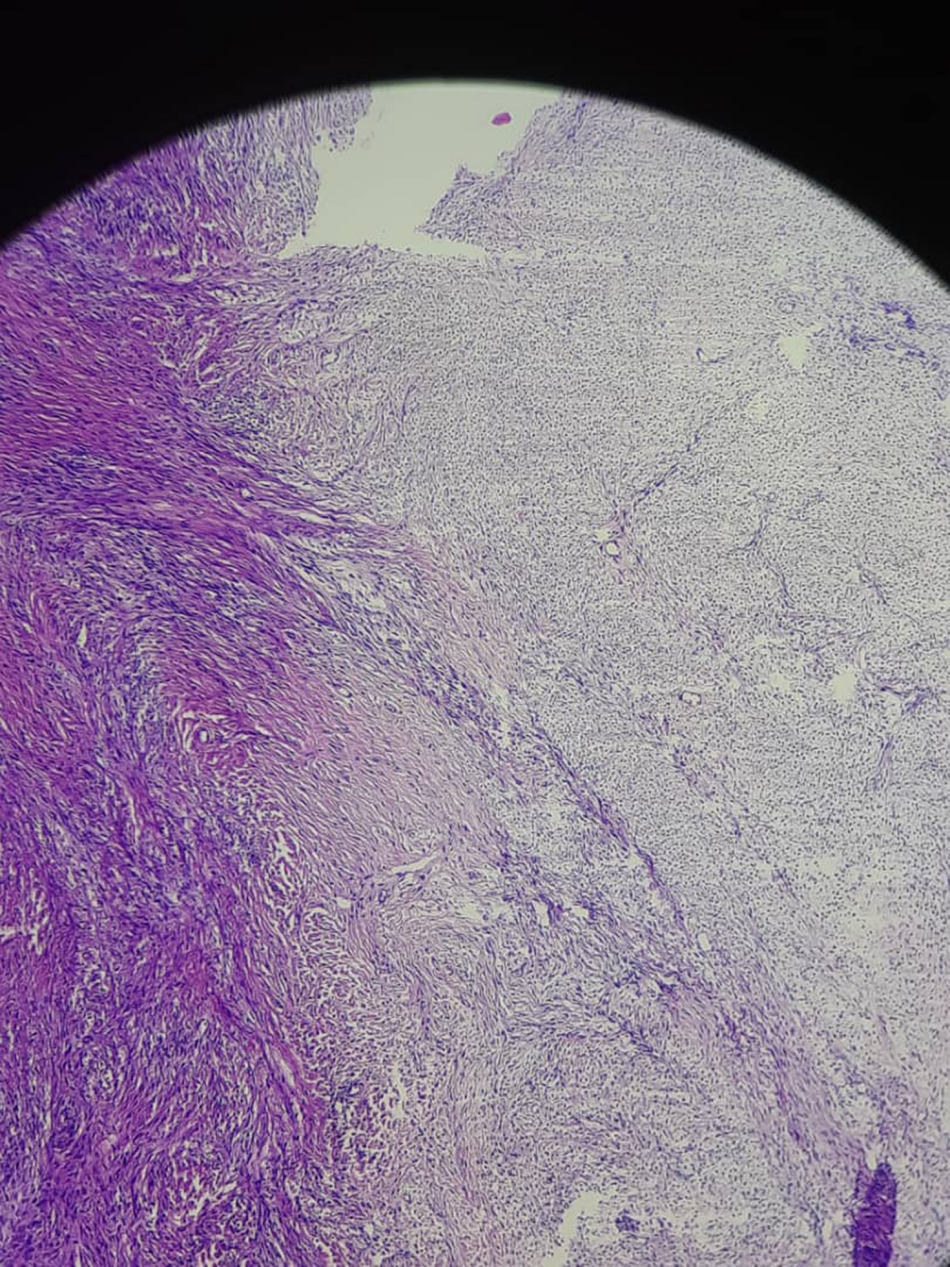
Histopathology; sections display ovarian tissue with well‐demarcated diffuse proliferation of cells with ovoid to round nuclei and pale cytoplasm. Cellular atypia and mitotic activity is absent. Extensive areas of necrosis and hemorrhage were also observed.

On pathological examination, the specimen was reported as deformed right ovary with tumoral invasion. Sections revealed diffuse proliferation of cells with pale cytoplasm, round to ovoid nuclei without nuclear atypia and absent mitotic figures (Figure 3). The findings were reported to be suggestive of thecoma with torsion.

The patient had no postoperative complication, and ultrasound was performed after 3 days which demonstrated a fetus with normal heart rate and a weight of 206 grams. Gestational age was 18 weeks and 2 days based on biparietal diameter and head circumference, and the amount of amniotic fluid was normal. The patient was discharged from the hospital after the ultrasound and follow‐up along with progesterone treatment and supplements were recommended.

## 3 DISCUSSION

Ovarian thecoma is a commonly benign low incidence rate tumor (0.15% to 1% of all ovarian tumors) arising from the ovarian stroma. Clinical manifestations are not very much specific from other ovarian tumors and preoperative diagnosis is challenging, as it may cause abdominal pain, abdominal distension, and pelvic mass. These tumors rise in postmenopausal women and are rare before the age of 40. Most of thecoma lesions are unilateral, various in size, shape, and even pathologic features as they may coexist with other sex cord‐stromal tumors like fibroma and granulosa cell tumors. Nearly, one‐third of these tumors can manifest hormonal production such as estrogen with clinical presentations such as abnormal menstrual bleeding, virilization, menstrual irregularities, and even infertility. Thecoma and fibromatous tumors can also be associated with ascites and sometimes pleural effusion.^2^, ^5^, ^6^, ^7^

In the present case, the main complaint was abdominal pain, and pelvic fluid was noticed in ultrasound examination, in addition to the ovarian torsion. However, the patient's emergency situation made it impossible to assess other clinical and paraclinical manifestations.

The general sonographic features of ovarian thecoma‐fibroma group are adnexal masses, which are hypoechoic with well‐demarcated borders and acoustic attenuation. There are minimal Doppler flow signals unless a complication like malignancy or torsion is present. Most of the time, the tumor is unilateral with a diameter smaller than 5 cm. The tumor diameter is reported to be significantly correlated with CA125 level and the volume of ascites fluid.^1^

The present case was not diagnosed with an ovarian tumor until the increase in tumor size along with the enlargement of the uterus led to torsion and acute pain. Ultrasound demonstrated the whirlpool sign of ovarian torsion, which is characterized by the appearances of a twisted ovarian pedicle.^8^ In a recent study by Jie‐Ling Feng et al. in 2020, sonographic and pathological findings of ovarian torsion were compared in pregnant and non‐pregnant women. The authors stated that ovarian edema, blood flow alteration, and the whirlpool sign were all reliable ultrasound markers. They also reported that preoperative ultrasonic detection rate of ovarian torsion was higher in pregnant women. The underlying lesions were more likely to be benign in pregnant women and more frequently malignant in non‐pregnant women.^9^ Although most ovarian cystic masses observed in pregnancy are corpus luteum cysts which regress spontaneously, it is mandatory to be extra careful when dealing with solid masses and to consider cautious radiological examination and continuous monitoring of the patient. Surgery should be offered when cancer is suspected or when there is a high risk of adverse complications, including ovarian torsion.^10^

In terms of pathologic examination, microscopic examination of thecoma usually demonstrates a primarily diffuse growth with varying degrees by hyaline plaques, nodular pattern, and calcification. Forty percent of the tumors are reported to have a fibroma component. The differential diagnosis is predominantly with other sex cord‐stromal neoplasms, particularly other stromal tumors, steroid cell tumor, and adult granulosa cell tumor.^11^

Fertility‐sparing surgery is the general approach for sex cord‐stromal tumors, as they tend to present in young patients with early stage disease. Complete surgery (total abdominal hysterectomy and removal of the other ovary) is considered in women older than child‐bearing age, and chemotherapy is reserved for advanced disease or recurrence.^4^, ^12^ It is recommended for all women with thecoma to undergo pre or intraoperative endometrial sampling in order to rule out endometrial hyperplasia or carcinoma, but since our patient was pregnant, the “hands off the uterus” rule was followed.^3^, ^4^ Although the present patient conservatively underwent tumor mass resection, a unilateral salpingo‐oophorectomy has been suggested by some experts in sex cord‐stromal tumors that present in young patients.^13^ Given the benign nature of thecoma and the rarity of pelvic and para‐aortic nodal involvement, lymph node sampling can be omitted for patients with stage I disease.^14^ However, careful follow‐up, and endometrial sampling at the earliest opportunity, is recommended in similar patients.

Ovarian sex cord‐stromal tumors, especially thecomas, are rare complications in pregnancy. The behavior of the disease, the clinical course, and fetomaternal outcomes are not fully understood in such cases. Blake et al. performed a systematic review on fetomaternal outcomes of pregnancies complicated by ovarian sex cord‐stromal tumors. The most common histologies were granulosa cell tumor (22.0%), thecoma (18.6%), and Sertoli–Leydig cell tumor (8.5%), respectively. The majority of cases resulted in successful live birth, with most cases being benign localized tumors which underwent unilateral adnexectomy. Despite the favorable results mentioned above, these conditions are considered high‐risk pregnancy. Risk factors such as younger age (<30 versus ≥30), large tumor (size ≥15 cm versus <15 cm), and stage (higher than I) increase the chance of adverse events.^15^

Considering the specific diagnosis of thecoma in pregnancy, reported experiences in literature are merely case reports. Okada et al. reported thecoma in a pregnant woman in first trimester which presented with abdominal distention due to ascites, and elevated serum CA125 levels. The patient underwent ascites aspiration and unilateral adnexectomy.^2^ The increase in CA125 levels, which was not observed in our patient, is considered to be a secondary reaction to hyperergasia of the peritoneal mesothelium caused by peritoneal stimulation. It is noteworthy that in the case reported by Okada et al., this stimulation of the peritoneum also manifested itself as ascites. Fetal growth and outcome were satisfactory during the follow‐up, which were similar to our patient. The authors also mentioned the 17 historical patients with pregnancy complicated with thecoma, which were reported in a case series by Hopkins et al, and emphasized on differentiating between benign tumors and malignant tumors through careful pathological examination when facing an ovarian solid tumor.^2^, ^16^ An exactly similar case to the present patient was reported in 2007 in a 19‐week pregnant woman with similar treatment and outcome.^17^ Other even more infrequent reports are a case of bilateral ovarian thecoma, and a synchronous Stump Appendicitis and Ovarian Fibro‐Thecoma, both diagnosed in pregnancy and managed with safe fetomaternal outcomes.^18^, ^19^

## 4 CONCLUSION

Thecoma is a predominantly benign tumor of ovary, which belongs to sex cord‐stromal tumors' category. Ovarian torsion in pregnancy can occur due to an ovarian thecoma. Although most tumors of this group are safely treated with fertility‐sparing surgery and have favorable fetomaternal outcomes similar to the present case, it is important to differentiate the lesions from malignant tumors through careful pathological examination and consider evaluation of risk factors, which are reported to be associated with worse outcomes.

## AUTHOR CONTRIBUTION

A.M. and M.K. contributed to conception, design, and drafting of the manuscript. Authors A.M. and A.z.M. contributed to data collection.

## CONFLICT OF INTEREST

The authors have no conflict of interest to declare.

## ETHICAL APPROVAL

Written informed consent was obtained from the patient to report their case, and the manuscript was approved at the Ethics Committee of Mashhad University of Medical Sciences.

## CONSENT

Published with written consent of the patient.

## Data Availability

All data generated during this study can be accessed through direct communication with the corresponding author and the agreement of all research team members.
